# The Role of Combination Therapy with α-Blockers and Hexanic Extract of *Serenoa repens* in the Treatment of LUTS/BPH

**DOI:** 10.3390/jcm11237169

**Published:** 2022-12-02

**Authors:** Cosimo De Nunzio, Andrea Salonia, Mauro Gacci, Vincenzo Ficarra

**Affiliations:** 1 Department of Urology, Sant’Andrea Hospital, Sapienza University of Rome, 00185 Rome, Italy; 2 Department of Urology, University Vita-Salute San Raffaele, 20132 Milan, Italy; 3 Division of Experimental Oncology, Unit of Urology, Urological Research Institute, IRCCS Ospedale San Raffaele, 20132 Milan, Italy; 4 Department of Minimally Invasive and Robotic Urologic Surgery and Kidney Transplantation, Careggi University Hospital (AOUC), University of Florence, 50134 Florence, Italy; 5 Department of Human and Pediatric Pathology “Gaetano Barresi”, Urologic Section, University of Messina, 98122 Messina, Italy

**Keywords:** lower urinary tract symptoms, benign prostate hyperplasia, hexanic extract of *Serenoa repens*, combination therapy

## Abstract

The hexanic extract of *Serenoa repens* (HESr) has been in use for decades as an effective, safe and well-tolerated therapy for relieving bothersome lower urinary tract symptoms (LUTS) associated with benign prostate hyperplasia (BPH). This manuscript gives an overview of HESr as monotherapy for LUTS/BPH treatment and focuses on the currently available literature investigating the possible clinical benefits of HESr combination therapy with α-blockers. Combination therapy of HESr with α-blockers has been gaining significant interest in recent years, as an increasing body of evidence shows the beneficial pharmacological effects that HESr treatment can add to standard first-line treatment with α-blockers. By reducing persistent Prostatic Inflammatory Status (PIS), commonly present in LUTS/BPH patients, HESr complements the relaxation of prostate smooth muscle induced by α-blockers, thus providing additional symptom relief. Data suggest that patients harbouring PIS and having a specific clinical profile might especially benefit from the combination therapy. Future therapeutic efforts may take advantage of more personalised strategies for LUTS/BPH management.

## 1. Introduction

Lower urinary tract symptoms (LUTS) associated with benign prostate hyperplasia (BPH) is a highly prevalent urological disease, particularly in aging men [1]. LUTS includes voiding symptoms (e.g., hesitancy, intermittency, slow stream, splitting or spraying, straining, terminal dribble) and storage symptoms (e.g., nocturia, urgency, increased frequency, urinary incontinence). These bothersome symptoms can interfere with daily activities and have a significant negative impact on the quality of life (QoL) of patients and their partners [2,3]. 

Despite its high prevalence and significant socio-economic burden [4], the pathogenesis of LUTS/BPH is not fully understood. Its multifactorial aetiology includes age-related prostatic tissue remodelling, hormonal alterations and the concomitant presence of metabolic syndrome (MetS). These multiple factors affect not only the prostate but also the bladder and urethra. Prostatic inflammation is a common histopathological finding in tissues obtained by prostate biopsies, trans-urethral resection or simple prostatectomies. In the REDUCE clinical trial, prostate biopsy showed chronic inflammatory cells and other histological signs of persistent Prostatic Inflammatory Status (PIS) in 77.4% of men with LUTS/BPH [5]. PIS is also associated with the severity and progression of the disease [6,7,8,9]. In addition, prostatic inflammation has been suggested as a connecting link between MetS and BPH [10]. Patients who have increased insulin resistance, hypertension and hypercholesterolaemia may have persistent PIS and are at increased risk for LUTS/BPH development [11]. Targeting prostatic inflammation has been recently considered a promising new therapeutic approach for relieving LUTS/BPH symptoms [12].

The dominant role of the androgen system and androgen receptor in the pathophysiology of LUTS/BPH is well defined, although underlying mechanisms are still not clear [13]. It is widely accepted that enhanced expression of α1-adrenoceptors and exaggerated α1-adrenergic smooth muscle tone in the hyperplastic prostate cause bladder outlet obstruction and LUTS. Commonly used medical treatments for LUTS/BPH therefore include α1-adrenoceptor antagonists (α1-blockers) and 5-alpha-reductase inhibitors (5-ARIs) as a first-line treatment in daily clinical practice [14]. α1-blockers provide fast relief of the obstructive LUTS by inducing relaxation of prostate smooth muscle, while 5-ARIs reduce androgenic stimulation of the prostate, resulting in epithelial atrophy and reduction in prostatic volume with time. 

Although α1-blockers are the most commonly used drug to manage LUTS/BPH, several combination treatments have been proposed and recognised in the EAU guidelines, including the combination with 5-ARIs or muscarinic receptor antagonists [1]. 

Hexanic extract of the American dwarf palm tree *Serenoa repens* (HESr) is another widely used treatment option to relieve LUTS/BPH symptoms and recently included in the EAU guidelines. A combination of α1-blockers and phytotherapy has been reported as the most commonly prescribed combination treatment among French general practitioners [15]. There is an increasing interest in evaluating the possibility to use HESr in combination with other drugs for managing LUTS/BPH. However, the currently available literature and clinical studies are limited. This review aims to summarise current evidence regarding the impact of HESr in combination with α-blockers on LUTS/BPH and to identify possible indications and future directions of this therapeutic approach. 

## 2. Materials and Methods

**Figure 1:**
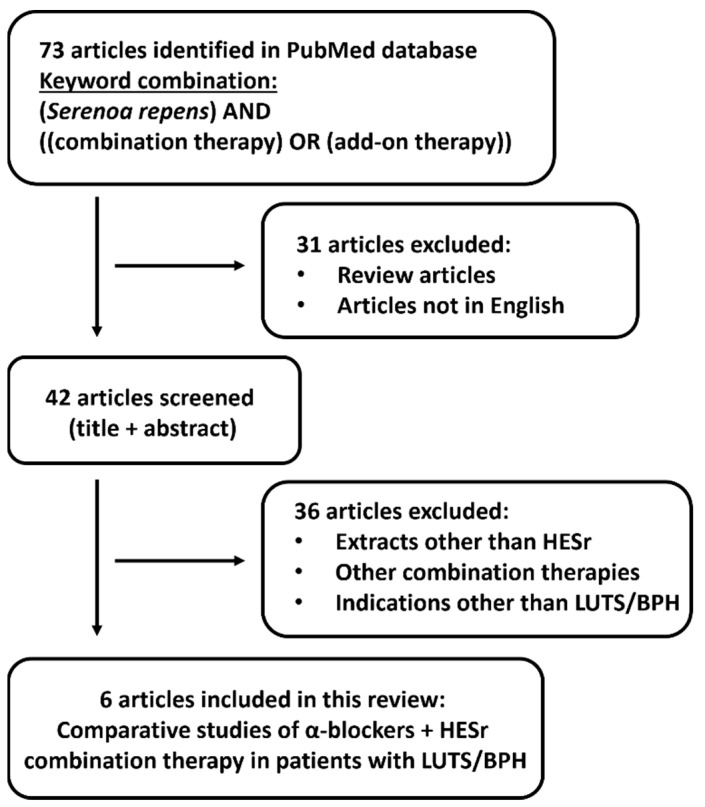
Flowchart of the study selection process. HESr—hexanic extract of *Serenoa repens*. LUTS/BPH—lower urinary tract symptoms associated with benign prostate hyperplasia.

A review of the literature for original articles in the English language on *Serenoa repens* combination therapy with α-blockers published/e-published up to October 2022 (no date restriction) was performed using the National Library of Medicine’s PubMed database (Figure 1). Keywords used for the systematic search included:

- (*Serenoa repens*) AND ((combination therapy) OR (add-on therapy))

Review articles and non-English language original studies were excluded from the initial search. The abstracts of the retrieved published records were screened by authors to identify and read the most relevant articles which were defined as comparative studies including patients treated with a combination of α-blockers and HESr. Studies using *Serenoa repens* extracts other then HESr or in indications other than LUTS/BPH as well as studies of combination therapies other than *Serenoa repens* in combination with α-blockers were excluded. Randomised and non-randomised clinical studies were selected, while studies published only as abstracts and meeting reports were not included in the review. Although the literature search was done in a systematic way, not all data sources were used. The selection of references was not all-inclusive and selection bias may have occurred.

## 3. Overview of HESr Monotherapy in the Management of LUTS/BPH

Extracts of *Serenoa repens* have been traditionally used for relieving the symptoms of LUTS/BPH for many decades. Similar to other herbals, many different extracts of the plant are available on the market. Different extraction processes result in significant qualitative and quantitative differences in the content and potency of bioactive compounds [16,17,18,19]. This is causing considerable variations in the clinical effects of each extract brand and creates controversy about the efficacy of HESr. In some studies where combined data of different brands were evaluated together, *Serenoa repens* extract appeared to have the same effect as placebo in relieving LUTS/BPH symptoms [20,21,22]. Similarly, a recent network meta-analysis found no clinically meaningful improvement with *Serenoa repens* extracts (hexanic and non-hexanic) versus placebo and α-blockers in men with LUTS after a short-term follow-up [23]. However, these conclusions might have been influenced by methodological limitations [24] as treatment with *Serenoa repens* extracts showed a clinical benefit after a longer treatment period (12 months). Additionally, HESr showed a greater improvement in the International Prostate Symptom Score (IPSS) than non-hexanic extracts. On the other hand, studies using only HESr demonstrated similar efficacy of this type of extract in relieving symptoms and improving QoL as α1-blockers and 5-ARIs, with fewer adverse effects [25,26,27,28]. Two meta-analyses focusing only on HESr found that HESr treatment reduced nocturia and improved maximum urinary flow rate (Qmax) compared to placebo and had a similar favourable effect on LUTS as the α-blocker tamsulosin and short-term 5-ARIs [29,30]. In addition, a recent paired matched clinical study demonstrated greater improvements of symptoms and QoL in patients treated with HESr versus watchful waiting [31]. The body of published clinical evidence therefore needs to be interpreted with caution. Based on the current evidence, only the hexanic extract of *Serenoa repens* is recommended by European Medicines Agency (EMA) as a medicinal product with recognised efficacy and acceptable safety for the treatment of LUTS/BPH [1,32]. 

**Figure 2:**
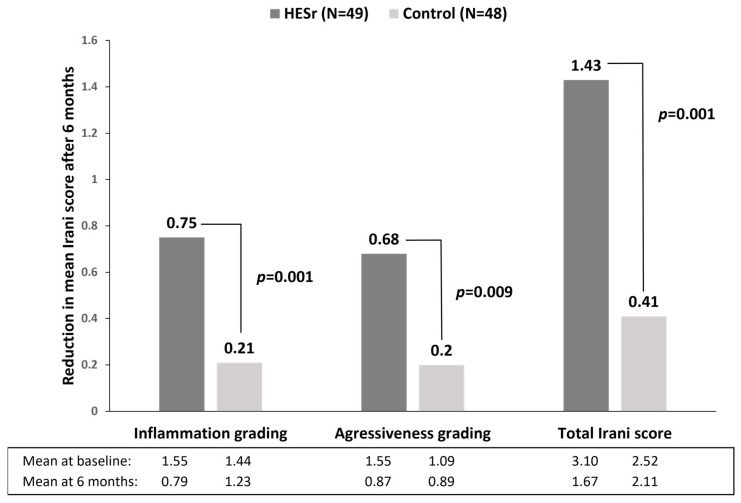
Histopathological findings according to Irani’s score at baseline (first prostate biopsy) and after 6 months (second prostate biopsy) [36]. The Irani score classifies prostatic inflammation on a 4-point scale based on the extension of inflammatory cells and their effect on prostate tissue. HESr—hexanic extract of *Serenoa repens*.

HESr has been shown to have anti-inflammatory, anti-androgenic and anti-proliferative activity [33,34,35]. Recent studies have demonstrated that HESr relieves LUTS by reducing underlying prostatic inflammation [12,36,37]. In a randomised clinical trial including 97 patients with histologically confirmed prostatic inflammation on prostate biopsy, 6 months of treatment with HESr (320 mg/day) significantly improved Irani’s inflammation grading, aggressiveness and total score in a second prostate biopsy done after the treatment (Figure 2) [36]. Moreover, immunohistochemical analysis showed a significant decrease in infiltrated inflammatory cells (T- and B-lymphocytes and macrophages). This finding is of particular importance for patients at increased risk of having persistent PIS, since patients with LUTS/BPH and persistent PIS not only have a higher risk of progression but also lower response rates to medical therapy [5,6,7].

## 4. HESr as a Combination Therapy with α-Blockers in the Management of LUTS/BPH

To date, several studies have investigated the potential benefit of combination therapy with HESr and α-blockers in comparison to α-blockers alone or in combination with 5-ARIs in men with LUTS/BPH (Table 1). One prospective, randomised study found that addition of *Serenoa repens* extract to α-blockers did not provide any clinical benefits in terms of IPSS score or Qmax in patients with LUTS/BPH [38]. However, this study did not include detailed information about the type of *Serenoa repens* extract used which may influence the quality and potency of the compound. In addition, the study design, a small patient population or a short follow-up time could also have led to significant bias in the study.

**Figure 3:**
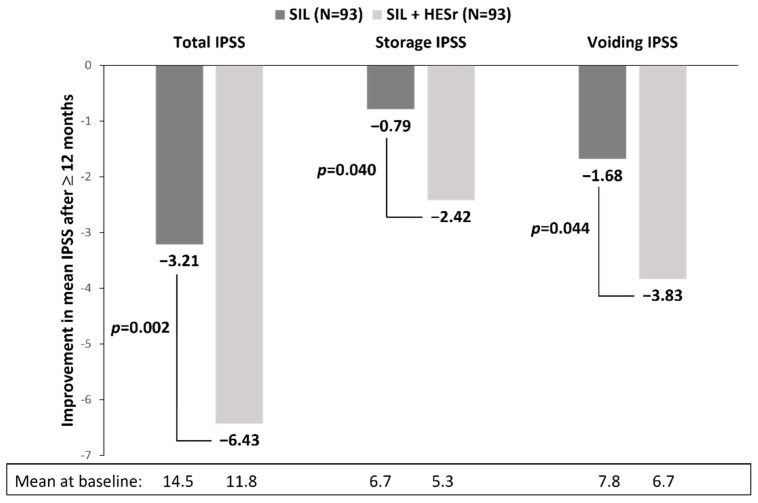
Improvement in mean IPSS after ≥12 months of treatment with 8 mg/day of α-blocker silodosin (SIL) alone or in combination with 320 mg/day of HESr (SIL + HESr) in 186 men with LUTS/BPH [40]. HESr—hexanic extract of *Serenoa repens*; IPSS—International Prostate Symptom Score; SIL—silodosin.

An open-label, randomised, Korean study involving 103 LUTS/BPH patients showed that 1 year of treatment with a combination of tamsulosin (0.2 mg/day) and HESr (320 mg/day) was as effective as tamsulosin monotherapy in reducing total and voiding IPSS [39]. In addition, combination therapy resulted in a greater improvement in the storage IPSS (1.9 versus 0.9 points, *p* = 0.021). Reported adverse events were similar between the two treatment groups, with slightly lower frequency of ejaculatory disorders reported by the patients receiving the combination treatment [39]. An Italian cross-sectional, matched-pair study compared silodosin monotherapy (8 mg/day) with a combination of silodosin and HESr in 186 LUTS/BPH patients treated for ≥12 months [40]. The mean improvement in total IPSS, including both the voiding and storage IPSS component, was significantly greater in patients receiving combination therapy (6.43 points) compared to those receiving silodosin alone (3.21 points, *p* = 0.002) (Figure 3). The improvement in Qmax was also greater with combination therapy (4.3 versus 2.3 mL/s), but not significantly (*p* = 0.15).

A longitudinal, prospective, observational multicentre QUALIPROST (Quality of Life in Benign Prostatic Hyperplasia) study showed that 6 months of HESr treatment had a similar effect on symptoms and patient QoL as α-blockers and 5-ARIs, with fewer adverse effects [25]. A subset analysis of the QUALIPROST study investigated the effect of HESr combination therapy with α-blockers on patient QoL [41]. Tamsulosin + HESr produced a greater symptom relief and a greater improvement in QoL than either treatment alone, with an acceptable tolerability profile. Analysis of data from 709 patients with moderate-to-high symptoms of LUTS/BPH demonstrated the greatest improvement in the combination treatment group for QoL (*p* < 0.02) and IPSS (7.2 points, compared to 5.7 points with tamsulosin alone and 5.4 points with HESr (*p* < 0.001)). Adverse effects were reported by 1.9% of patients receiving HESr, 13.3% receiving tamsulosin, and 12.0% receiving tamsulosin with HESr (*p* < 0.001). The most common adverse event was ejaculation disturbance, which was reported less frequently by patients receiving combination therapy versus tamsulosin alone (11.4, 8.2 and 0.8% in tamsulosin, combination therapy, and HESr treatment group, respectively).

**Figure 4:**
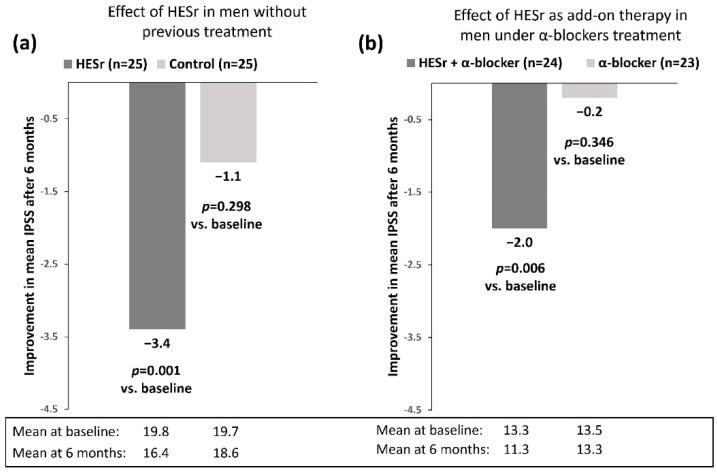
Improvement in mean IPSS after 6 months of HESr therapy (320 mg/day) in men without previous treatment (**a**) and men under treatment with α-blockers (**b**) [37].

A post hoc analysis of a randomised biopsy study [36] evaluated the clinical impact of 6 months of treatment with HESr (320 mg/day) on patients with LUTS/BPH and confirmed prostatic inflammation and investigated potential baseline parameters that may be associated with a better response to treatment [37]. The study population included 110 patients without any previous treatment for LUTS/BPH or under treatment with α-blockers at baseline. The impact of HESr treatment was investigated in these two sub-populations. IPSS improved statistically significantly with HESr as monotherapy or as add-on therapy to α-blockers, while it remained stable in both control groups (Figure 4). Moreover, a fair correlation was found between prostate volume and reduction of inflammation in patients treated with HESr. Additionally, a stronger Qmax improvement was found in patients with a specific clinical profile such as higher BMI, larger prostate volume and presence of diabetes mellitus, suggesting a better treatment response in these patients. Thus, HESr combination therapy with α-blockers should be considered as a favourable personalised treatment option for this patient population.

Another subset analysis from the QUALIPROST study was recently published and is the first clinical study up to date comparing the effects of α-blockers in combination with HESr or 5-ARIs in patients with moderate-to-severe LUTS/BPH [42]. Paired matched analysis showed that 6-month treatment with tamsulosin + HESr led to similar levels of improvement in symptoms and QoL as treatment with tamsulosin + 5-ARIs, though with considerably fewer side effects (10.3% versus 26.5% in tamsulosin + HESr and tamsulosin + 5-ARIs treatment group, respectively) [42]. Erectile dysfunction, reduced libido, and anejaculation were the most frequent adverse events reported by the patients receiving tamsulosin + 5-ARIs. Despite relatively low numbers of patients included in this retrospective subset analysis, the value of this last study is that the analysed data were collected under conditions of current clinical practice reflecting a real-world situation.

## 5. Discussion

The almost endemic prevalence of LUTS/BPH in the aging population gives rise to the need for a treatment option that provides clinically meaningful improvements and has a favourable safety profile. A standard therapeutic approach with α-blockers and 5-ARIs may be efficient in improving symptoms and urinary flow. However, these drugs may also have significant side effects such as hypotension, gynecomastia and sexual dysfunction (e.g., decreased libido, erectile dysfunction and/or ejaculatory disorders) [43,44], especially when used in combination [45,46]. As LUTS/BPH aetiology is multifactorial, combining drugs with different modes of action might have advantages over the monotherapeutic approach. There is increasing knowledge on pathways of non-adrenergic prostate contraction induced by activation of receptors other than α1-adrenoceptors that could explain the limited effects of α-blockers monotherapy [47]. Simultaneous targeting of α1-adrenergic and non-adrenergic contractions of the prostate opens up possibilities for efficient combined therapy approaches influencing prostate growth and contraction on multiple levels. Furthermore, there is growing evidence that α-blockers have limited efficacy in patients with PIS [6,12].

**Figure 5:**
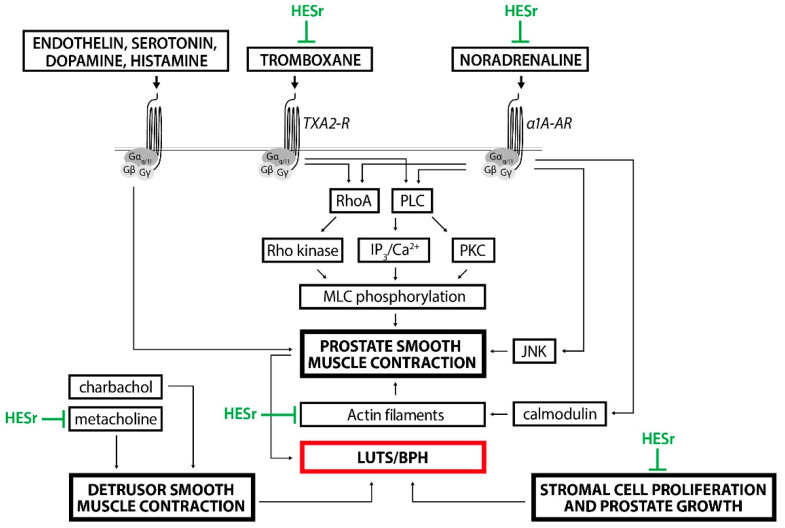
Mechanisms of HESr action in the management of LUTS/BPH. HESr inhibitory effects (green) on different targets and shared intracellular mediators contribute to the LUTS/BPH symptom relief on multiple levels. Modified from [47,48]. TXA2-R—tromboxane A2 receptor; α1A-AR—α1A-adrenoreceptor.

HESr is a well-tolerated and widely prescribed treatment option for patients with LUTS/BPH. In recent years, several clinical trials have shown the benefit of HESr in combination with α-blockers for the management of LUTS/BPH. Multicomponent action of HESr complements the blockade of α1-adrenoceptors with other mechanisms of action. The anti-inflammatory effects of HESr are particularly important for patients with PIS. Data show that by reducing the underlying persistent PIS, HESr provides additional LUTS/BPH symptom relief when combined with α-blockers, including improvements in the voiding and storage IPSS and urinary flow rate. Furthermore, a recent publication has shown that HESr may inhibit neurogenic, α1-adrenergic and thromboxane-induced smooth muscle contraction in the prostate tissue as well as methacholine-induced contractions of the bladder detrusor smooth muscle in vitro [48]. Additionally, HESr treatment inhibited prostate stromal cell growth and actin formation [48]. These results provide evidence that can explain the ubiquitously beneficial effects of HESr reported by patients and seen in clinical trials and give rationale for HESr combination approach with α-blockers (Figure 5).

In summary, HESr combination with α-blockers showed similar efficacy to α-blockers alone or in combination with 5-ARIs [25,42]. Importantly, combination treatment induced significantly fewer side effects than α-blocker monotherapy, particularly those associated with sexual function [25]. This is of clinical relevance since most men prefer lower-risk management options that have fewer sexual side effects and are aimed at improving storage symptoms such as urgency, incontinence and nocturia [49]. Accordingly, this should be taken into account when counselling patients, together with patient expectations and risk-benefit preferences. In the real-world setting, combination therapy of HESr with α-blockers is frequently adopted as a first-line therapy strategy providing improvements over either therapy alone, as shown by a cross-sectional study of representative consulting patients with LUTS/BPH in two European countries [50]. Data show that patients prescribed HESr combination therapy with α-blockers were more likely to be older and retired than those prescribed monotherapy. They were also less likely to be newly diagnosed with LUTS/BPH. The key drivers of HESr combination therapy choice were reported as clinical symptom alleviation, strong association with the lack of sexual dysfunction risk and reduction of inflammation [50]. In addition, patients with PIS and a specific clinical profile tend to show a better treatment response and should be targeted for combination treatment [37]. The general clinical condition and comorbidities of the patient should be carefully evaluated in order to personalise the treatment and choose the right therapy for the right patient.

Available studies on the HESr/α-blocker combination approach have some limitations. There is a general lack of randomised clinical trials on this subject in the current literature. Studies often have a short follow-up or a low number of patients included, do not clearly define the type of *Serenoa repens* extract used or do not indicate whether monotherapy or combination therapy is used. To date, limited or no data are available from studies investigating other HESr combination therapies or which α-blocker is the most effective when used in combination with HESr. Considering the detrimental influence of LUTS/BPH on QoL and the positive outcomes of currently available studies, there is a clear need for more clinical evidence on combination therapies. The authors hope that the data summarised in this review would help in designing future randomised clinical trials as a step forward towards effective, personalised HESr/α-blocker combination treatments.

## 6. Conclusions

HESr in combination with α-blockers provides a greater symptom relief and fewer adverse events (including sexual dysfunction) in patients with LUTS/BPH than α-blockers alone, together with amelioration of underlying chronic prostatic inflammation. This is demonstrated by a growing number of studies showing significant improvements in both IPSS scores and patient perception of LUTS. Combination therapy of HESr with α-blockers could therefore be an effective therapeutic strategy, especially for patients with moderate-to-severe LUTS/BPH symptoms interested in preserving their sexual function. In addition, this combination therapy may be particularly recommended for patients with specific characteristics, such as a higher BMI, a larger prostate volume and diabetes mellitus, who seem to have a better treatment response. Well-designed randomised clinical trials in the future will contribute to our knowledge on the benefits of HESr combination therapy with α-blockers and help translate these findings into personalised treatments in everyday clinical practice.

## Figures and Tables

**Table 1:** Key features of included studies investigating HESr as a combination therapy with α-blockers in the management of LUTS/BPH.

Study	Study Design	Arms (*n*)	Mean Age at Baseline (SD)	Follow Up	Change in IPSS from Baseline, Mean (SD)	Change in QoL Score, Mean (SD)	Change in Q_max_ mL/s, Mean (SD)
Hizli and Uygur (2007) [38]	Prospective, randomised	nHESr (20)	56.8 (7.8)	6 mo	6.1 (2.7)	2.6 (0.9)	3.2 (2.2)
		Tam (20)	58.9 (5.7)	6 mo	4.6 (3.3)	2.1 (0.8)	3.7 (2.6)
		Tam + nHESr (20)	60.2 (6.3)	6 mo	4.9 (2.3)	2.2 (1.0)	4.2 (2.5)
Ryu et al. (2015) [39]	Prospective, randomised, open-label	Tam (53)	63.4 (1.4)	6 mo	4.4 (0.4)	2.0 (0.3)	1.8 (0.2)
				12 mo	5.5 (0.5)	2.5 (0.4)	2.0 (0.3)
		Tam + HESr (50)	62.5 (1.2)	6 mo	4.7 (0.3)	1.9 (0.2)	2.0 (0.3)
				12 mo	5.8 (0.4)	2.4 (0.4)	2.1 (0.3)
Boeri et al. (2017) [40]	Retrospective, non-randomised, cross-sectional	Sil (93)	57.9 (11.3)	13.5 mo	3.2 (0.6)	0.2 (0.2)	2.3 (0.4)
		Sil + HESr (93)	55.3 (12.2)	13.5 mo	6.4 (0.6)	1.0 (0.2)	4.3 (0.5)
Alcaraz et al. (2020) [41]	Retrospective, non-randomised, open-label	HESr (262)	64.6 (8.9)	6 mo	5.4 (4.6)	1.3 (1.3)	3.1 (4.2)
		Tam (263)	65.4 (8.0)	6 mo	5.7 (4.3)	1.3 (1.2)	2.9 (3.8)
		Tam + HESr (184)	65.1 (8.0)	6 mo	7.2 (5.0)	1.8 (1.2)	2.0 (2.8)
Samarinas et al. (2020) [37]	Post hoc, randomised, blinded	Control (25)	68.7 (NR)	6 mo	1.1 (NR)	NR	1.5 (NR)
		HESr (25)	71.4 (NR)	6 mo	3.4 (NR)	NR	0.3 (NR)
		α-blocker (23)	68.7 (NR)	6 mo	0.2 (NR)	NR	0.2 (NR)
		α-blocker +HESr (24)	71.4 (NR)	6 mo	2.0 (NR)	NR	0.3 (NR)
Alcaraz et al. (2022) [42]	Retrospective, paired matched	Tam + HESr (68)	67.9 (7.9)	6 mo	6.7 (5.0)	1.7 (1.2)	1.6 (1.7) *
		Tam + 5-ARI (68)	68.3 (7.3)	6 mo	7.7 (6.3)	1.7 (1.3)	2.2 (5.6) *

HESr—hexanic extract of *Serenoa repens*; nHESr—type of extract of *Serenoa repens* not clear; Tam—tamsulosin; Sil—silodosin; 5-ARI—5-alpha-reductase inhibitor; IPSS—International Prostate Symptom Score; QoL—the quality of life; Qmax—maximum urinary flow rate; *n*—number of patients; SD—standard deviation; NR—not reported; * Number of patients vary—data not available for all patients.

## Data Availability

The authors confirm that the data supporting the findings of this study are available within the corresponding articles.
